# Size and frequency of natural forest disturbances and the Amazon forest carbon balance

**DOI:** 10.1038/ncomms4434

**Published:** 2014-03-18

**Authors:** Fernando D.B. Espírito-Santo, Manuel Gloor, Michael Keller, Yadvinder Malhi, Sassan Saatchi, Bruce Nelson, Raimundo C. Oliveira Junior, Cleuton Pereira, Jon Lloyd, Steve Frolking, Michael Palace, Yosio E. Shimabukuro, Valdete Duarte, Abel Monteagudo Mendoza, Gabriela López-González, Tim R. Baker, Ted R. Feldpausch, Roel J.W. Brienen, Gregory P. Asner, Doreen S. Boyd, Oliver L. Phillips

**Affiliations:** 1 NASA Jet Propulsion Laboratory, California Institute of Technology, Pasadena, California 91109, USA; 2 Institute for the Study of Earth, Oceans and Space, University of New Hampshire, Durham, New Hampshire 03824, USA; 3 School of Geography, University of Leeds, Leeds LS2 9JT, UK; 4 USDA Forest Service, International Institute of Tropical Forestry, San Juan 00926-1119, Puerto Rico; 5 EMBRAPA Monitoramento por Satélite, Campinas, Sao Paulo CEP 13070-115, Brazil; 6 Environmental Change Institute, School of Geography and the Environment, University of Oxford, Oxford OX1 3QY, UK; 7 National Institute for Research in Amazonia (INPA), CP 478, Manaus, Amazonas 69011-970, Brazil; 8 EMBRAPA Amazônia Oriental (CPATU), Santarém, Pará CEP 68035-110 C.P. 261, Brazil; 9 Belterra, Pará CEP 68143-000, Brazil; 10 Centre for Tropical Environmental and Sustainability Science (TESS), School of Earth and Environmental Sciences, James Cook University, Cairns, Queensland 4878, Australia; 11 National Institute for Space Research (INPE), São José dos Campos, Sao Paulo CEP 12227-010, Brazil; 12 Jardin Botanico de Missouri, Oxapampa 19231, Pasco, Peru; 13 Department of Global Ecology, Carnegie Institution for Science, Stanford, California 94305, USA; 14 School of Geography, University of Nottingham, University Park, Nottingham NG7 2RD, UK; 15 Present address: College of Life and Environmental Sciences, University of Exeter, Rennes Drive, Exeter EX4 4RJ, UK

## Abstract

Forest inventory studies in the Amazon indicate a large terrestrial carbon sink. However, field plots may fail to represent forest mortality processes at landscape-scales of tropical forests. Here we characterize the frequency distribution of disturbance events in natural forests from 0.01 ha to 2,651 ha size throughout Amazonia using a novel combination of forest inventory, airborne lidar and satellite remote sensing data. We find that small-scale mortality events are responsible for aboveground biomass losses of ~1.7 Pg C y^−1^ over the entire Amazon region. We also find that intermediate-scale disturbances account for losses of ~0.2 Pg C y^−1^, and that the largest-scale disturbances as a result of blow-downs only account for losses of ~0.004 Pg C y^−1^. Simulation of growth and mortality indicates that even when all carbon losses from intermediate and large-scale disturbances are considered, these are outweighed by the net biomass accumulation by tree growth, supporting the inference of an Amazon carbon sink.

## Abstract (web summary)

The world’s tropical forests represent a terrestrial carbon sink, yet its size is uncertain. Espírito-Santo *et al.* characterize full Amazon disturbances combining forest inventories and remote sensing data, and use statistical modelling to quantify the Amazon aboveground forest carbon balance.

## Graphical abstract

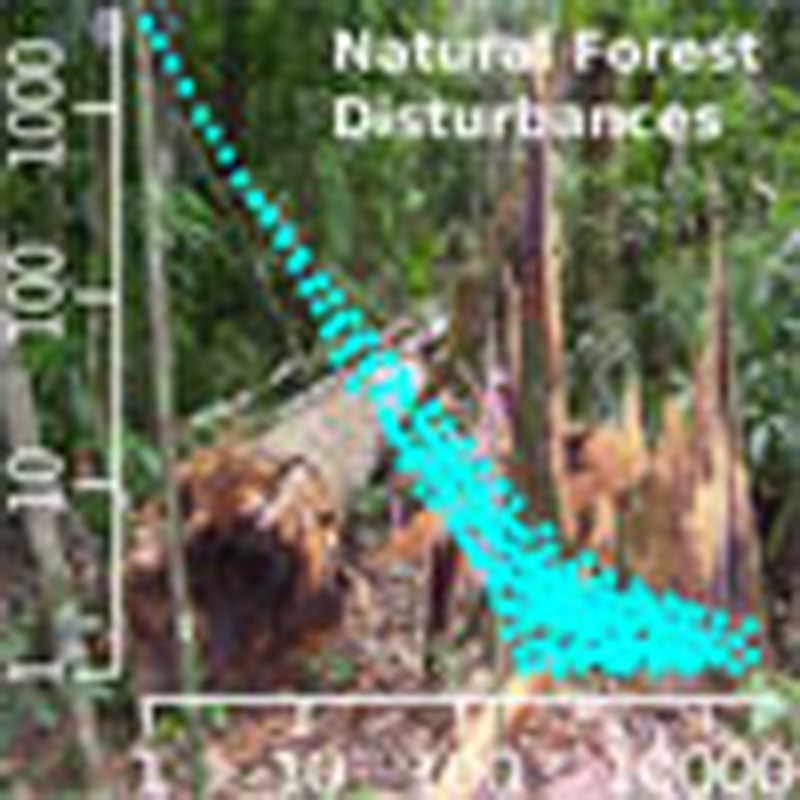

Global records of atmospheric CO_2_ concentrations, fossil fuel emissions and ocean carbon uptake, estimated on the basis of ocean surveys, indicate that there is a large terrestrial carbon sink^1^,^2^ of which a substantial portion may be due to uptake by old growth tropical forests^3^. On the other hand, were some of the current large tropical forest carbon pools (including ~100 Pg C in aboveground biomass (AGB) in Amazonia^4^,^5^) to be released rapidly to the atmosphere^6^, it would substantially enhance greenhouse warming^1^. Understanding the nature and trajectory of the Amazon forest carbon balance is therefore of considerable importance.

The evidence for a tropical old-growth forest sink^7^,^8^ is based primarily on repeated biometric measurements of growth and death of individual trees across the tropics. These measurements indicate that at the plot-level old-growth forests in Amazonia and Africa have apparently gained carbon over the last 30 years^8^,^9^,^10^,^11^. The extrapolation of regional trends from a relatively small number of ~1-ha-sized plots has been questioned because potentially undersampled natural disturbances at the landscape-scale could counterbalance tree level growth^12^,^13^. Thus, according to this view, forest plots are biased towards those parts of the landscape recovering from natural disturbance.

Resolving this issue requires assessing whether estimates of biomass gain are robust when fully considering disturbances of all sizes^14^,^15^; here we test this statistically against the null hypothesis of net zero change in biomass. We synthesize and characterize the frequency distribution of natural disturbance at all spatial scales across forests of the Amazon region using a combination of forest censuses, airborne lidar and passive optical remote sensing from satellites (Fig. 1). We ask whether the net biomass gains inferred from forest census data are an artefact of the small size and limited number of plots in the plot network^10^. We address this question using a simple stochastic forest simulator based on growth statistics from the forest census network and the new regional disturbance size-frequency distribution scaled to all Amazon forest regions. We find that large-scale blow-downs and medium-sized disturbances have a minor impact on AGB change of South American tropical forests. Moreover, taking into account the full range of natural disturbances, we find support for the inference of a carbon sink in natural Amazonian forests.

## Results

### Amazon-wide frequency distribution of natural forest disturbances

There are two spatially disjoint size and severity domains of large disturbances in the Amazon: one domain with large blow-downs centred west of Manaus and another large one where the largest blow-downs are absent (Fig. 2). Although it has been suggested that the disturbance size frequency distribution follows a power law *p*(*x*)∝*x*^−α^ (probability density *p*(*x*) and size of an event *x*)^16^, the observed distribution suggests a more subtle picture (Fig. 3). Visually three sections may be identified: an approximately exponential decline of frequency with size for smallest size disturbances, a power law-type decline for intermediate scales and another power law decline for the largest-scale disturbance blow-downs (Fig. 3a,c). The power law decline for intermediate disturbances with size appears to be steeper than for largest blow-downs. This is seen in the estimated return intervals versus disturbance severity relationship (see Methods) that reveals a sharp increase to higher values for intermediate range disturbances from 1 to 10 ha (Fig. 3b,d). The data also show that disturbance-induced tree mortality that cause small-area disturbances have a return interval of ~100 years (Fig. 3b,d). This agrees with studies from other tropical forest regions that observed an annual tree-fall disturbance rate of 1% by the process of gap formation due to tree death^17^. By contrast, the return interval of large blow-downs is very long—that is, such events are extremely rare—ranging from 4 × 10^5^ year to >10^7^ year depending on size (Fig. 3b). Small disturbances (<0.1 ha) per year are many orders of magnitude more frequent (~10^10^ events) than large blow-downs (~10^3^ events) over the Amazon (Fig. 3a).

### Forest biomass loss from disturbance

**Figure 1:**
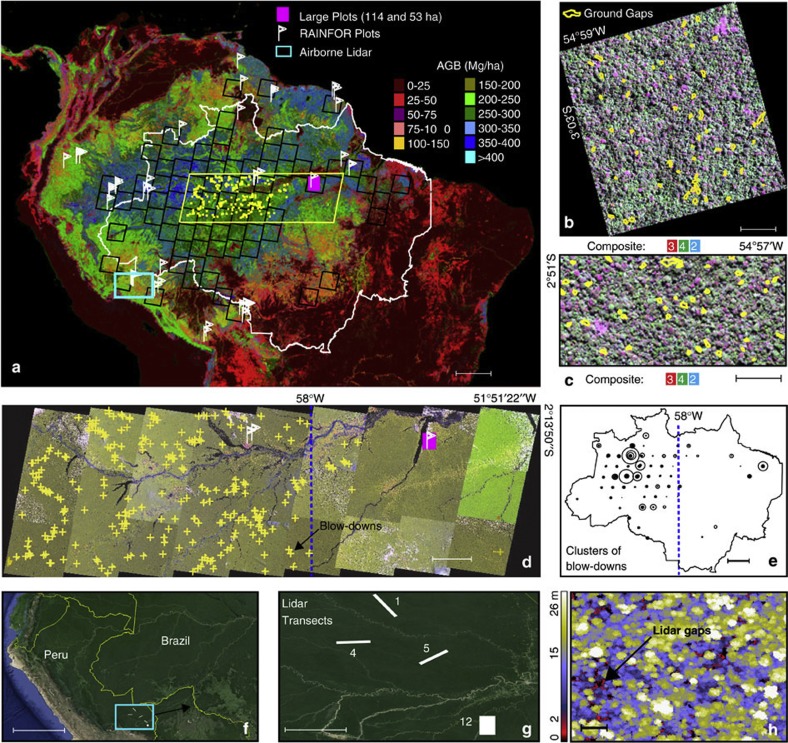
Amazon Basin-wide data of natural forest disturbances. (**a**) Spatial distribution of RAINFOR forest census plots^10^ (*n*=135), inspected Landsat images (*n*=137) with occurrences of large blow-down disturbances ≥30 ha (ref. 26) (black boxes, *n*=330 blow-downs) and ≥5 ha (ref. 25) (yellow dots, *n*=279 blow-downs) underlain by an ABG map of the Amazon. White, yellow and turquoise in (**a**) indicate the Brazilian border, the mosaic of Landsat images in the Central Amazon^25^ (as shown in (**d**)), and the lidar airborne campaigns in Peru^24^, respectively. (**b**) Large forest inventory plot of 114 ha (ref. 23) with ground gaps (yellow polygons, *n*=55) overlain on a high-resolution IKONOS-2 image acquired in 2008 in the Eastern Amazon. (**c**) Large plot of 53 ha (ref. 23) with ground gaps (*n*=51) over a second high-resolution IKONOS-2 image acquired in 2009. (**d**) Digitally classified blow-downs in an East-West mosaic of Landsat images from the Central Amazon. (**e**) Representation of disturbance size areas found in all Landsat images—blow-downs disturbances ≥30 ha areas are proportional to the size of the circles. (**f**) Location of the lidar airborne campaigns in the Southern Peruvian Amazon^24^ (turquoise box). (**g**) Lidar data collections in four large transects of tropical forest (48,374 ha, *n*=30,130 gaps ≥20 m^2^ in erosional *terra firme* and depositional forests). (**h**) Details of the detection of gaps in lidar canopy height model (CHM)—a 2 m height threshold was used to detect tree-fall gaps in CHM (**h**). Composite in (**b**) and (**c**) means colour compositions of IKONOS-2 image at full-width wavelength for three bands: (2) green 0.51–0.60 μm, (3) red 0.63–0.70 μm and (4) NIR 0.76–0.85 μm. Dashed blue lines in Landsat images (**d**) and central Brazilian Amazon (**e**) divides the areas with high frequency of blow-downs (≥5 ha) between 58°00′W and 66°49′W (western Amazon) and where blow-downs are infrequent in the eastern basin (51°51′W to 58°00′W). Legends of scale-bar for all areas (**a**–**h**) are 500km, 0.2km, 0.2km, 90km, 500km, 572km, 45km and 0.5 km, respectively.

Based on the size and frequency of natural disturbances of our data (Fig. 1, Supplementary Fig. 1 and Supplementary Table 1) scaled to the entire Amazon forest area (~6.8 × 10^8^ ha)^18^, the total carbon released by natural disturbances is estimated as 1.88 Pg C y^−1^, where ~1.66 Pg C y^−1^ or ~88.3% is accounted for by small-scale mortality (<0.1 ha), ~12.7% from intermediate (0.1–5 ha) and ~0.02% from large disturbances (≥5 ha). Large disturbances although visually impressive are extremely rare (Fig. 3b,d), and the estimated amount of biomass loss is only 0.004 Pg C y^−1^. By comparison net carbon emissions caused by forest clearing in the Brazilian Amazon^19^ in the 1990s were ~0.2 Pg C y^−1^. Conversion of the mortality to Amazon forest areas implies that natural mortality affects 2.0 × 10^7^ ha y^−1^ or 2% of total forest area, where ~80.0% is from small-scale mortality, ~19.9% is from intermediate and only 0.1% from large disturbances.

### Implications of natural forest disturbances for the carbon cycle

The estimated disturbance spectrum permits us to address whether the observed carbon balance of the Amazon tropical forests inferred from forest plot censuses does indeed statistically significantly reflect carbon gain (carbon accumulation rates significantly greater than zero). For this purpose we use a stochastic forest growth simulator^10^ of the form *dM*=*G* × *dt*–*D × dt*, where *dM* is aboveground forest biomass loss in units of carbon per area, d*t* a time interval, here 1 year, and *G* and *D* stochastic variables distributed according to the observed distributions of aboveground mass gain due to growth (*G*) and loss (*D*) due to mortality^10^,^12^,^20^ (for details please see Methods). We use the simulator to assess the mean net carbon balance and its s.d. We simulated 10^9^ equivalent annual observations of each scenario and statistical significance of the results is assessed using a *t*-test (Table 1). The scenario that we consider to be most realistic for the whole Amazon region is marked bold in Table 1. For all scenarios ensemble mean net gains are positive and for all but the most extreme scenario, the *t*-tests reveal significance. Intermediate disturbances have a notable effect on the mean but relatively small effect on the variance. In contrast, large disturbances have no perceptible effect on the mean but greatly increase the variance. The exceptional scenario—which given our data are clearly over-pessimistic—assumes both that the largest blow-downs occur not only in Central Amazonia but throughout the Amazon forest regions, and that intermediate disturbances occur at a rate that greatly over-represents the importance of fluvial disturbances (Supplementary Table 2).

## Discussion

In summary, we have characterized the full size distribution and return frequency of natural forest mortality and disturbance in the Amazon forest biome (Fig. 3). Our findings help to resolve the debate about the relative importance of intermediate- and larger-area disturbances^10^,^12^,^13^,^20^ and gains in biomass stocks in tropical forest plots^10^ for determining the regional-scale carbon balance. In our simulation taking into account the full range of natural disturbances, we find significant increases in the biomass of Amazonian forest. Although the simulation does not consider the spatial and temporal interactions of growth and disturbance, these results nonetheless imply that natural disturbance processes in Amazonia are insufficiently intense or widespread to negate the conclusion from the pan-Amazon plot network that old-growth forests in that region have gained biomass. Uncertainty about the role of disturbances in affecting estimates of the long-term trajectory of the carbon balance of tropical forests is declining as the forest-monitoring effort on the ground increases both in time and space^8^,^9^,^10^,^21^. Our characterization of the natural disturbance regime of the Amazon forest yields new insight into the role of disturbance in tropical forest ecology and carbon balance.

## Methods

### Assessing the range of natural forest disturbances

Our approach includes natural causes of tree mortality^14^ (including partial mortality such as branch falls) that liberate carbon^6^, but excludes anthropogenic disturbance caused by forest clearing, logging and fires^1^,^19^. To determine the frequency distribution of natural disturbances in the Amazon at all scales, we quantify small-area disturbances using records of biomass losses from a long-term repeat measurement network spatially distributed across the entire Amazon^8^,^9^,^10^,^21^,^22^ supplemented by two large forest plot surveys (53 and 114 ha) in the Eastern Amazon^23^. We quantify intermediate-area disturbances using tree-fall gaps detected by airborne lidar from four large surveys (48,374 ha) in Southern Peru^24^, and large area disturbances from blow-downs using two data sets^25^,^26^ from Landsat satellite images in an East-West transect^25^ and over the entire Brazilian Amazon forest^26^ (Fig. 1).

**Figure 2:**
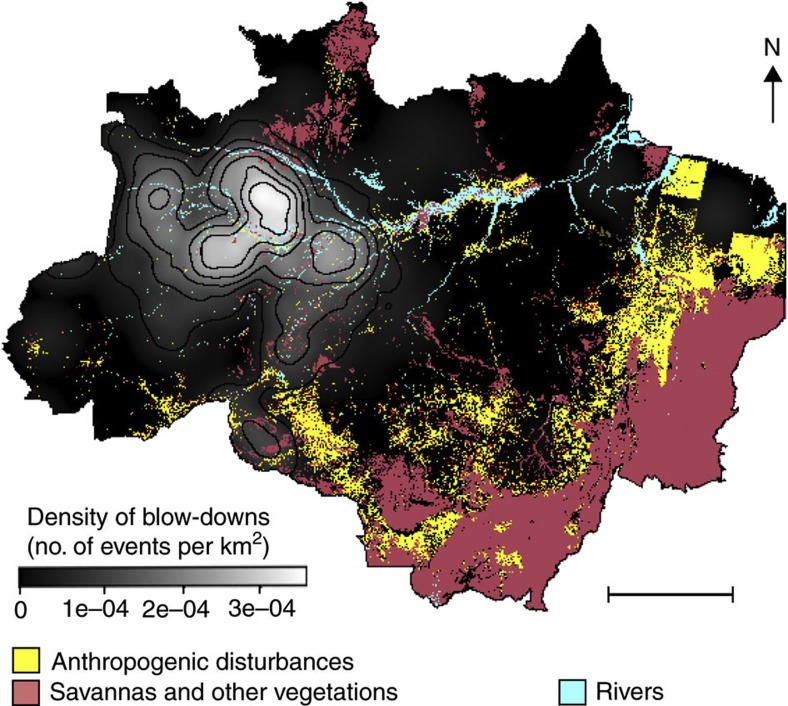
Spatial distribution of large disturbances in the Brazilian Amazon. Cluster map of blow-downs of Brazilian Amazon using a Gaussian smoothing kernel^28^ with bandwidth of 200 km modelled from 330 large disturbances ≥30 ha detected in 137 Landsat images over the Amazon region^26^. Colour bar is the intensity of large disturbances in the Amazon (number of blow-downs per km^2^). Legend of scalebar for the map of blow-down density is 500 km.

For small-area disturbances we estimate biomass loss associated with area loss of each event of disturbance. For intermediate disturbances several assumptions are required to translate the measurements of forest structure from ~1 m airborne lidar data into an estimate of biomass loss (Supplementary Figs 2 and 3). To ensure that our test of the hypothesis that the plot network effectively measures biomass change is as robust as possible for natural forests (Supplementary Fig. 4), our assumptions conservatively err on the generous side to the magnitude and frequency of intermediate disturbance. For large disturbances, we reanalyzed records of blow-downs likely caused by downdrafts associated with convective clouds^27^ covering Brazilian Amazon forests^26^ using historical Landsat satellite images (pixels sizes ~30 m) (Supplementary Fig. 5) and a more recent East–West mosaic of Landsat scenes covering a portion of the Amazon^25^ (Fig. 1d). Combining the spatial records of large disturbances detected by Landsat^25^,^26^ with a recently developed map of AGB^5^, we estimate carbon loss associated with these large disturbances (Figs 1d and 2). Because of the uncertainties associated with below-ground biomass^1^,^2^,^5^,^19^, we discuss carbon losses only in terms of AGB, which probably accounts for ~80% of live biomass in Amazonia^4^,^5^,^19^.

For determining the largest blow-downs we build on an earlier study of large natural disturbances^26^ that identified 330 blow-downs ≥30 ha distributed in 72 Landsat scenes from the total 137 scenes (Supplementary Fig. 5) acquired between 1988 and 1991 across the ~3.5 × 10^6^ km^2^ forested area of the Brazilian Amazon^18^. Subsequent digital image processing for blow-down detection^25^ in the Central Amazon collected around the year 2000 (27 Landsat scenes) further revealed a substantial number of medium-sized blow-downs (5–30 ha) not detected using earlier visual inspection methods^26^. In both studies^25^,^26^ spatial analysis showed a high concentration of all detected blow-down disturbances west of ~58° W clearly associated with areas of strong convective activity^27^ as measured from cloud-top temperatures from the TRMM satellite^25^. Reanalyzing that data^26^ here using a Gaussian kernel smoothing algorithm for cluster analysis^28^ confirms the concentration of blow-down disturbances in the western Amazon (Fig. 2) with blow-downs 12 times more frequent west of 58° W than to the east. This conclusion does not depend on the bandwidth size used for the cluster analysis (Supplementary Figs 6–8).

### Forest inventories and remote sensing to assess disturbance

Effective detection of forest disturbance that results in tree mortality^2^,^9^,^12^,^13^,^20^,^23^ and the release of carbon to the atmosphere^1^,^2^,^6^,^19^ requires observational methods that encompass relevant spatial scales^1^,^14^. We combine repeat measurements from forest censuses with analysis of Landsat and lidar data permitting us to estimate mortality across all relevant spatial scales (Fig. 1). For mortality that affects less than ~0.1 ha, we combine two spatial and temporal sources of data: (1) 484 forest plot censuses from 135 (~1 ha) permanent plots covering 1,545 census years of tree-by-tree measurements, distributed over the entire Amazon region including the Guiana Shield (see Supplementary Methods), from the RAINFOR network that covers 45 Amazon regions^10^ and (2) losses of biomass in areas of branch or tree-fall gaps^6^,^12^,^17^ of two plots of 53 and 114 ha from the Tapajós National Forest in the Eastern Brazilian Amazon^23^. To estimate disturbances at an intermediate area (from 0.1–5 ha) we used a large area of airborne lidar data from four samples in the Southern Peruvian Amazon^24^ covering in total 48,374 ha. For disturbances covering large areas (disturbance size ≥5 ha) we combine three remote sensing data sets: (1) a spatially extensive record of large disturbances from blow-downs ≥30 ha from 128 Landsat scenes from the Brazilian Amazon and 8 scenes from outside of Brazil^26^, (2) a high-resolution study of blow-downs ≥5 ha using 27 Landsat scenes on an east-west transect in the central Amazon^25^ and (3) a multi-sensor remote-sensing product of AGB for the tropics^5^. For all mortality (Table 1; Supplementary Fig. 9 and Supplementary Table 1) we estimate areas and biomass defined as losses in AGB stocks (Supplementary Fig. 3). For forest plot data we estimated area losses from biomass losses assuming a constant biomass density (see Supplementary Methods). We caution that this approach assumes that all biomass disturbances are linearly correlated with area of the disturbances, which is a rough approximation^14^.

### Return interval versus disturbance size

To estimate return intervals of forest area loss events of a given size we first scale-estimated number frequencies to the full Amazon forest by multiplying them with the ratio 
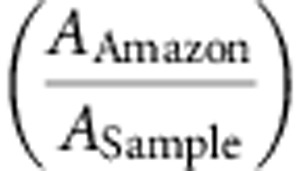
, where *A*_Amazon_ (6,769,214 km^2^; INPE^18^, Supplementary Fig. 4) is the total forest area of the Amazon and *A*_Sample_ is the sample area. The empirical probability p*(*A*)Δ*A* that a fixed location will be affected by a disturbance of area *A* during 1 year is then 
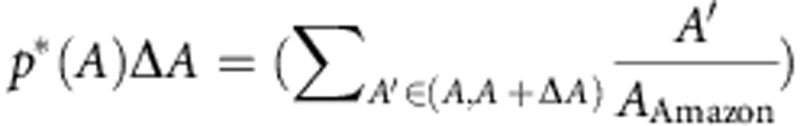
, where the sum is over all events across the Amazon region with area *A′* in the interval (*A*, *A+*Δ*A*) and Δ*A* is a finite area interval. The probability *P*(*A*_event_≥*A*) for the occurrence of a disturbance event per year with area loss larger than *A* at a fixed location is then 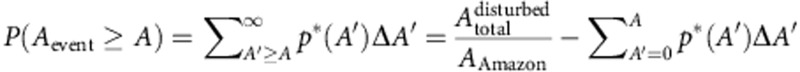 using the identity 
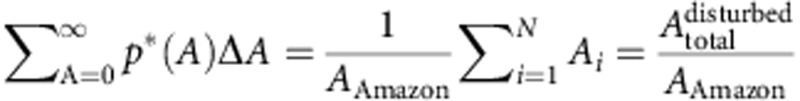
 (that is, not 1, therefore the notation p* instead of *p*), where *N* is the total number of observed disturbances and 
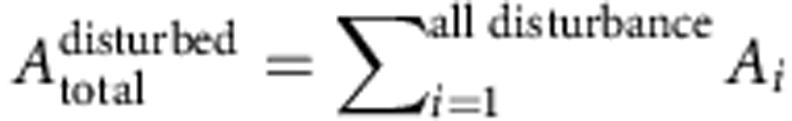
 is the total annually disturbed forest area in the Amazon. Therefore, an estimate for the return interval *τ*(*A*_event_≥*A*) of a disturbance event with forest area lost larger than *A*_event_ at a fixed location is given by the inverse of the probability of observing such an event per year: 
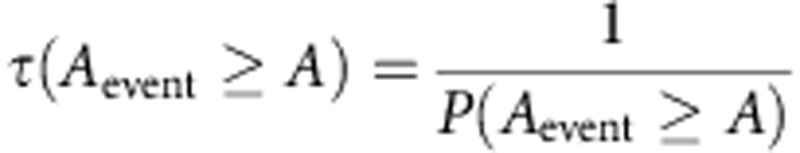
. An analogous equation holds for the return interval with respect to biomass loss associated with a disturbance event.

### Forest AGB simulation

**Figure 3:**
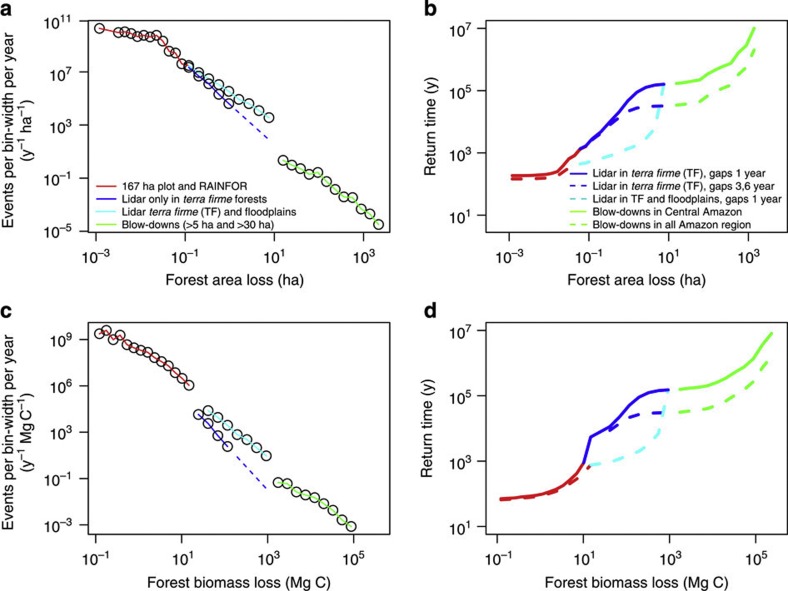
Estimated frequency distributions of natural forest disturbances in the Amazon. (**a**) Number of disturbances per year obtained by scaling observed events to the full Amazon region by multiplication with the inverse of observed area fraction. Number density of disturbances per year obtained from a histogram and dividing the resulting numbers by histogram bin-width. Bin-widths are chosen such as to include at least one event; the number density follows approximately Δlog (number of occurrences)/Δlog (disturbance size)≈−2.5. (**b**) Return intervals versus severity of events calculated using the inverse of the cumulative PDF (see Methods) for various combinations of the data from repeated plot measurements, lidar surveys and Landsat imagery. For (**a**) and (**b**) largest blow-downs (those detected by Landsat imagery) are scaled to the region by multiplication of Amazon area fraction with large blow-downs. Panels (**c**) and (**d**) are similar to (**a**) and (**b**) but with respect to disturbance biomass loss instead of disturbance area. In (**b**,**d**) solid lines correspond to the case where large blow-downs are included only in the Central Amazon while the dashed lines correspond to the case where largest blow-downs are assumed to occur everywhere in the region (as a sensitivity study) and similarly the dashed light blue line corresponds to the case where also floodplain lidar data with river-driven disturbances are included (note that the forest plot network is based overwhelmingly on non-floodplain plots).

Once the disturbance spectrum of AGB loss is defined we can infer the s.d. introduced into an ensemble of growth rates from forest censuses using the simple stochastic forest simulator of the form *dM*=*G* × *dt*–*D × dt* introduced above, which predicts forest carbon mass change per area (*M*) due to growth (*G*) and loss (*D*) due to mortality^10^,^12^,^20^ during the time interval d*t*. For *G* we used as input parameters growth from 484 forest censuses^10^ covering *N*=135 plots and *N*=1545 census years, and mortality (AGB loss) from our new disturbance spectrum analysis. To generate random numbers distributed according to our observed distribution we use the inverse transform method^10^. For growth we use specifically *G~N*(*μ*,*σ*) with *μ*=2.5 or 2.75 (Mg C ha^−1^ y^−1^), respectively, and *σ*=0.85 (Mg C ha^−1^ y^−1^), the mean value for the Amazon region according to the RAINFOR data^10^ and Eastern Amazon, respectively. For *D*, we used our Amazon forest mortality frequency distribution (Fig. 3) and modifications thereof for the purpose of sensitivity and uncertainty analysis of our approach (see main text and Table 1 and Supplementary Table 2). The growth component of the simulation model is conservative with respect to the hypothesis of net biomass gains, as it neglects the growth enhancement after disturbance events^29^ and so overestimates the period of biomass decline. In real forests, disturbance-recovery growth enhancements shorten the total period for which disturbance-induced net biomass losses occurs for any given patch of forest, and therefore mitigate the impact of disturbance events on the summary statistics of net biomass trajectories.

To ensure that the simulation of disturbances is operating correctly we checked the predicted Amazon disturbance spectrum against the observed spectrum using a sample of 5 × 10^8^ simulation runs, also revealing that such a number is sufficient to reproduce the full spectrum. The simulator was then run for 10^9^ annual equivalent samples for each scenario (Table 1, Supplementary Fig. 10, and Supplementary Table 2). We started the simulator from an arbitrary value zero and let mass accumulate or decline indefinitely thus, in effect, permitting us to represent the whole Amazon. From these 10^9^ samples of biomass gain or loss we assessed whether the inference of a large carbon sink in old-growth forests is statistically significant^10^, by consulting the *t* statistic 
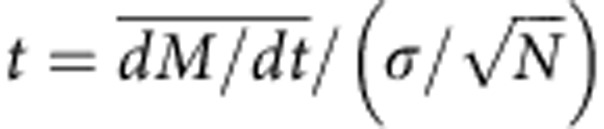
. 
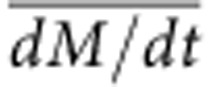
 is the trajectory sample mean net carbon balance over 1 year, and *σ* the trajectory sample s.d. over the same period. A *t*-test is justified given the large sample size despite the skewed distributions of net gains, that is, means are indeed nearly normally distributed as predicted by the central limit theorem and tested by Monte Carlo simulations based on the observed distribution of net gains.

We run the simulator for various disturbance distribution scenarios to explore the sensitivity of the model to parameter selection. Scenarios with results summarized in Table 1 include three blow-down extents (none, Central Amazon and the full region) and two assumed time-scales (1 and 3.6 year) for detectability of disturbances observed with lidar^24^. Sensitivity to change in growth rates and an extreme-case intermediate-disturbance regime taken from the Peruvian river floodplains are also examined (Supplementary Table 2). The two intermediate-disturbance area cases explore the sensitivity of our results to the spatially biased coverage of the lidar data to one part of the southwest Amazon. The first of the intermediate-scale scenarios use data from *terra firme* only, the most relevant data for answering our main question because *terra firme* forests occupy the overwhelming part of the Amazon region. The second extreme intermediate-scale scenario also includes lidar data from flooded forests, which have a greater frequency of larger area disturbance, presumably fluvially induced, although the effect of human disturbance cannot be categorically eliminated because the region studied is affected by extensive unregulated placer gold mining. For small- and large-area disturbances, we did not differentiate geomorphic regimes because they were not apparent in the data.

## Author contributions

M.K., M.G., O.L.P. and Y.M. conceived this study. F.D.B.E.-S., M.K., M.G., O.L.P. and Y.M. designed the research study. F.D.B.E.-S. integrated all data sets. F.D.B.E.-S. and M.G. calculated and analysed the data. M.G. created the stochastic simulator, ran the simulations and produced the regional frequency and return interval distributions. S.S. helped with blow-down carbon biomass losses. B.N. and F.D.B.E.-S. produced the regional map of blow-downs and the spatial analysis. F.D.B.E.-S., R.C.O.J. and C.P. collected the data of the large plots (114 and 53 ha) at Tapajós National Forest, Brazil. Y.E.S. and V.D. produced the remote sensing layers of undisturbed forest of South America. O.L.P., J.L., S.F., M.P. and all others authors helped with the review and suggestions. A.M.M., G.L.-G., T.R.B., T.R.F., R.J.W.B. and O.L.P. led and analysed more recent RAINFOR campaigns. G.P.A. provided and helped with the lidar data from Peru. F.D.B.E.-S., M.G., M.K. and O.L.P. wrote the paper.

## Additional information

**How to cite this article:** Espírito-Santo, F. D. B. *et al.* Size and frequency of natural forest disturbances and the Amazon forest carbon balance. *Nat. Commun.* 5:3434 doi: 10.1038/ncomms4434 (2014).

## Supplementary Material

Supplementary Information Supplementary Figures 1-10, Supplementary Tables 1-2, Supplementary Methods and Supplementary References

## Figures and Tables

**Table 1:** Summary of Amazon forest simulator results.

**Intermediate-scale disturbances**	**Large-scale blow-downs25,26**		
	**None**	**Central Amazon**	**All Amazon region**
*Lidar data*^24^ *from* terra firme *(gaps age*^30^ *~1-year old)*			
dM/dt* (Mg C ha^−1^ y^−1^)	—	0.85	—
σ* (Mg C ha^−1^ y^−1^)	—	4.40	—
* t*_obs_ (*N*=135)	—	2.24	—
* t*_obs_ (*N*=1,545)	—	7.59	—
			
*Lidar data*^24^ *from* terra firme *(gaps age*^30^ *~3.6-year old)*			
dM/dt* (Mg C ha^−1^ y^−1^)	0.94	**0.94**	0.94
σ* (Mg C ha^−1^ y^−1^)	2.19	**3.77**	12.4
* t*_obs_ (*N*=135)	4.99	**2.90**	0.88
* t*_obs_ (*N*=1,545)	16.9	**9.80**	2.98

Mean and statistical significance of simulated AGB gains for a range of scenarios. We vary occurrence of large-disturbance blow-downs^25^,^26^, that is, the large-end tail of the disturbance frequency distribution, and age of intermediate-range disturbances. For blow-downs we distinguish three cases: (i) no large-disturbance blow-downs^25^,^26^, (ii) large blow-downs as observed only in central Amazon (~20% of the Amazon region), (iii) everywhere in the Amazon with the same frequency of events as in the central Amazon (that is, with five times more large-area events than observed). For intermediate-range disturbances we distinguish disturbances occurring across the entire Amazon region distributed according to lidar surveys^24^ (plots 1, 4, 5 and 12) of erosional *terra firme* (ETF) forests (33,196 ha) with either a mean gap age of 1 or 3.6 years based on gap closure observations of a 50 ha plot on Barro Colorado Island^30^. We assumed an annual mean mass gain (*G*) (live tree mass gains plus mass gains due to recruitment^8^,^10^,^11^) of 2.5 Mg C ha^−1^ y^−1^ in areas of *terra firme* forests. The simulator of forest mortality (*D*) is based on the frequency distribution of disturbance area. To convert area losses to biomass losses we assumed a forest mass density of 170 Mg C ha^−1^ for all simulations, a high value and ~50% greater than the actual biomass density in the lidar landscape in southern Peru used to estimate intermediate disturbance dynamics^8^,^11^. Assessment of each scenario is based on a set of 10^9^ annual equivalent samples. The most credible results are in highlighted bold.

^*^Significance is assessed with a *t*-test considering *t*_sim_=(*dM/dt*)/(*σ*/sqrt(*N*)) where *dM/dt* is ensemble mean mass gain (Mg C ha^−1^ y^−1^), *σ* the s.d. of the mass gain distribution and *N* the number of observations. For *N* we use the RAINFOR sample published in 2009, either conservatively *N*=135, the total number of observational plots or *N*=1,545, the total number of plot census years, reflecting the stochastic nature of disturbance and therefore the near independence of plot results from year-to-year. Net gain results are statistically significant at the 95% level if *t*_sim_≥*t*_(0.975,*N*=135)_≈*t*_(0.975,*N*=1,545)_=1.96 and at the 99% level if *t*_sim_≥*t*_(0.995,*N*=135)_≈*t*_(0.995,*N*=1,545)_=2.58.
