# Supplementary material for: Size and frequency of natural forest disturbances and the Amazon forest carbon balance
Source: Nat Commun. 2014 Mar 18;5:3434. doi: 10.1038/ncomms4434 (PMC4273466; doi:10.1038/ncomms4434)
Supplement: Supplementary Information — Supplementary Figures 1-10, Supplementary Tables 1-2, Supplementary Methods and Supplementary References [file ncomms4434-s1.pdf]

Supplementary Information

Supplementary Figures

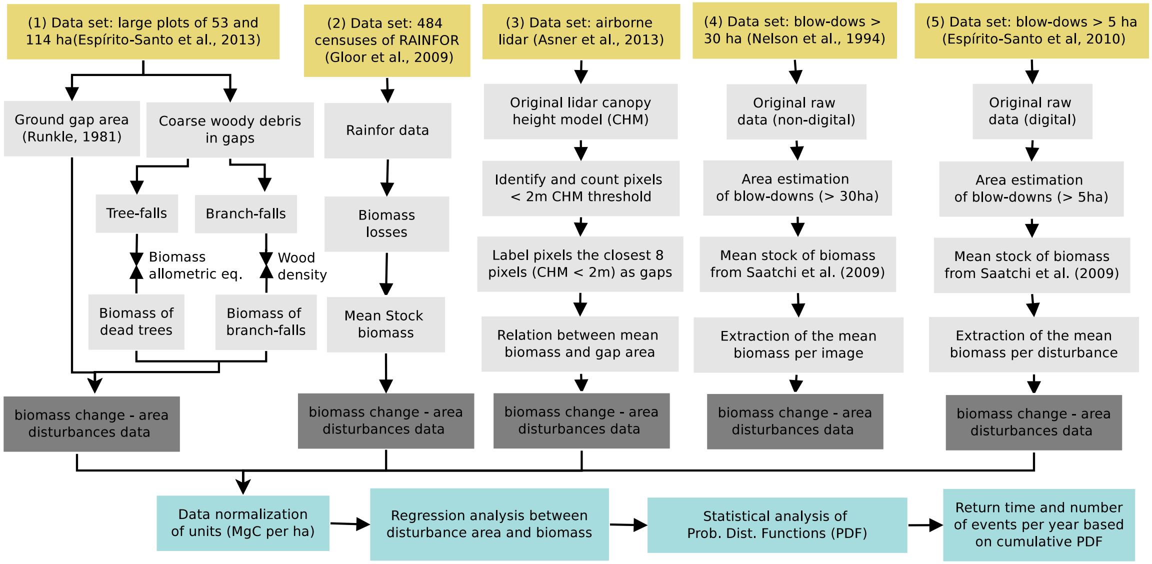

Supplementary Figure 1. *Schematic outline.* Main processing steps carried out to integrate several sources of disturbance data over the Amazon region

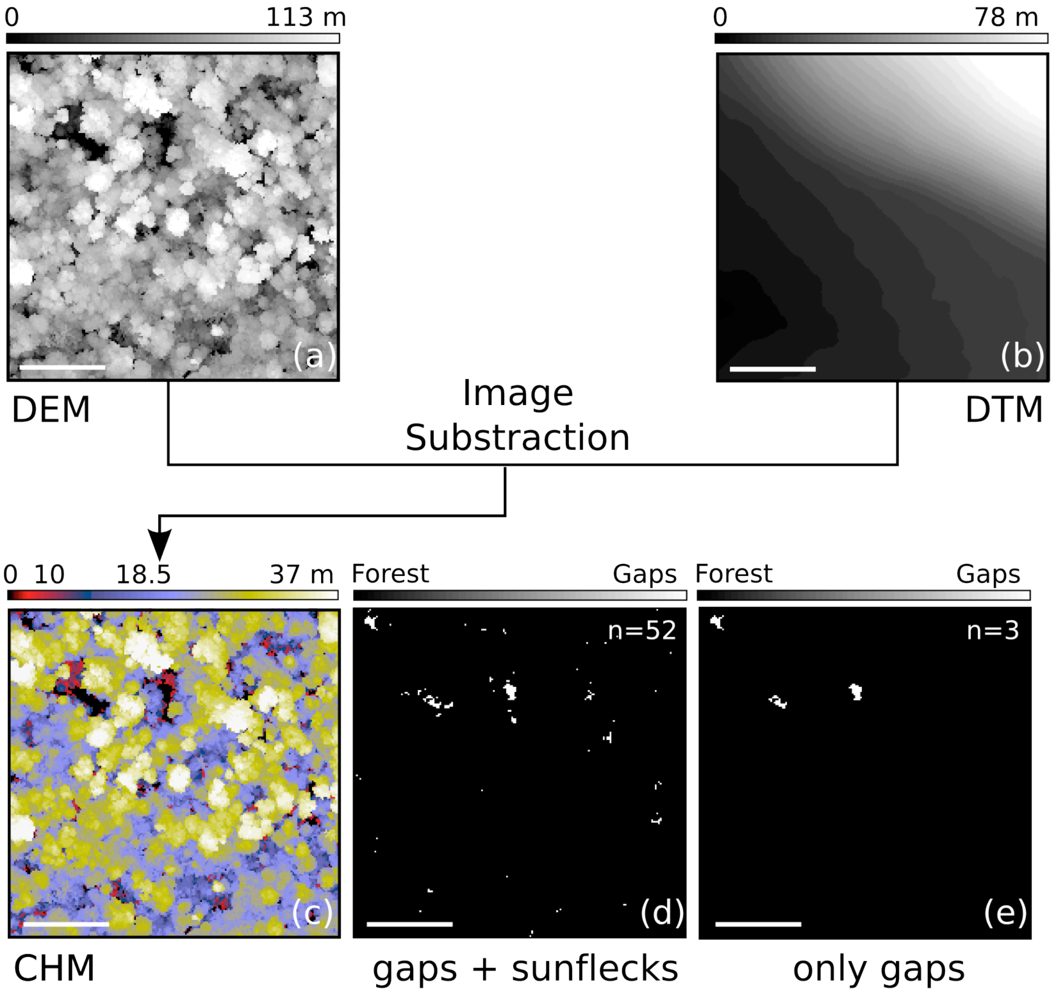

Supplementary Figure 2. *Example of image processing to extract and detect tree-fall gaps in lidar data.* (a) Digital canopy surface model and (b) digital terrain model were extracted from lidar cloud points to produce the (c) digital canopy model or tree height. (d) Forest sunflecks[31] (in this case 52 in number) detected by lidar were separated from (e) tree-fall gaps (in this case 3) using a minimum gap-size threshold of 20 m^2. Lidar grid image of 200 m by 200 m (4 ha).

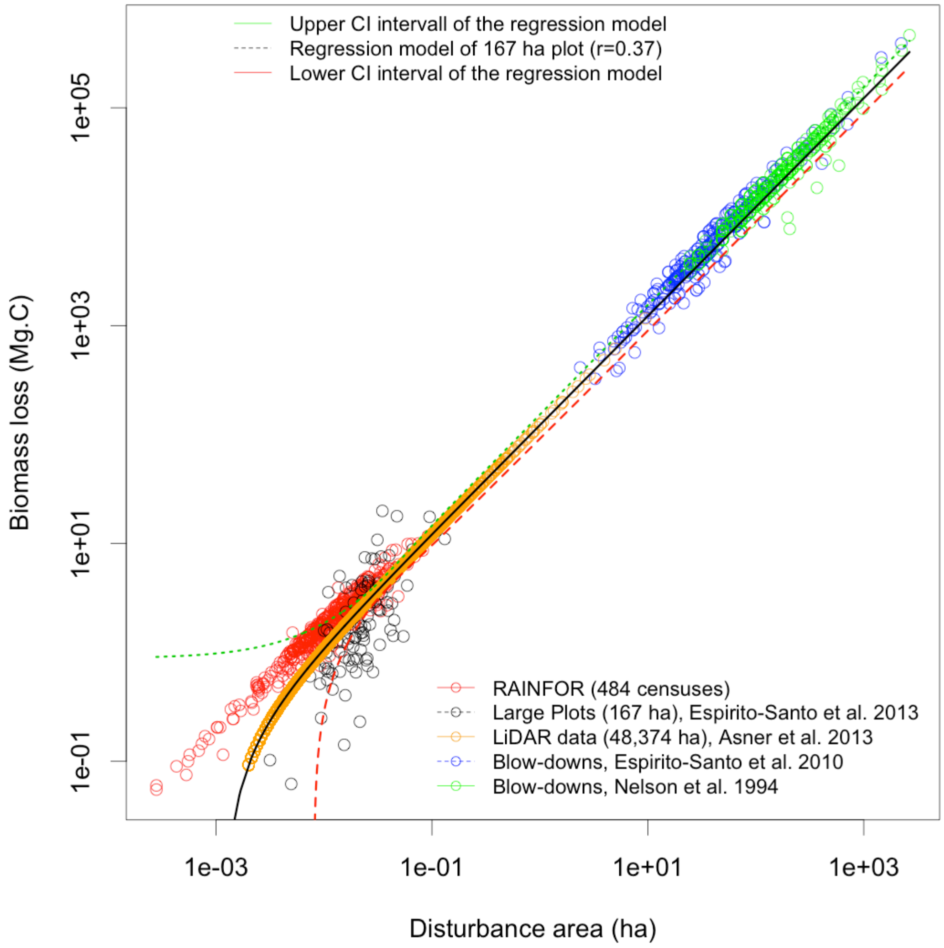

Supplementary Figure 3. *Relation between disturbance area and loss in aboveground biomass in the Amazon.* Data sets are from several studies of disturbances across the Amazon, from branch and tree falls to landscape scale blow-downs. Small disturbances: (1) in red, forest plot inventories (n=484 censuses of 135 * 1ha plots[10]) distributed over the Amazon and (2) in black, 96 tree-fall gaps from two large forest inventory plots (total area 167 ha) in the Tapajós National Forest[23]. Intermediate disturbances: (3) in orange, small and intermediate disturbances from 48,374 ha of lidar[24] data. Large disturbances: (4) in blue, 279 blow-downs bigger than 5 ha from an East-West mosaic of 27 Landsat scenes of the Amazon[26]; and (5) in green, 330 blow-downs greater than 30 ha from 136 Landsat scenes in the Brazilian Amazon[25]. A relation between area and biomass loss (Mg C) was tested from 96 tree-fall gaps (0.003 - 0.13 ha) where both area and aboveground biomass were measured. The linear regression fit is Biomass Loss = -0.1528 + 122.5073 (Disturbance Area) (n=96, r=0.37), in units of Mg C and ha for loss biomass and disturbance area, respectively.

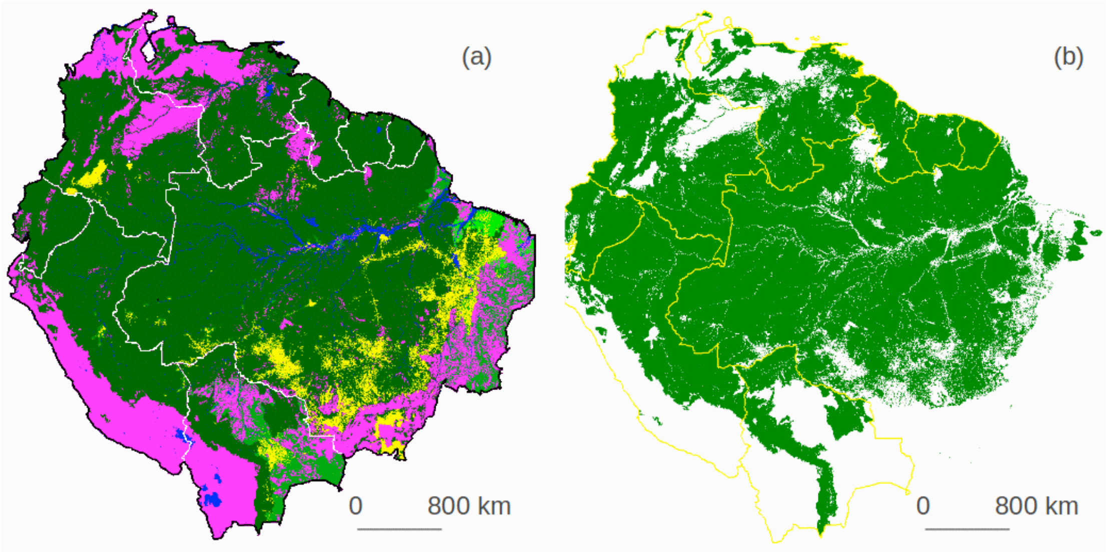

Supplementary Figure 4. *Amazonia land cover map.* (a) Map using historical Landsat and MODIS images from the Pan-Amazon project for the year 2010. (b) Undisturbed forests of tropical regions, excluding other types of land cover. Map colors represent the following categories: undisturbed forest (dark green), deforestation (yellow), savannas or/and grass vegetation (pink), secondary forest (light green) and water (blue).

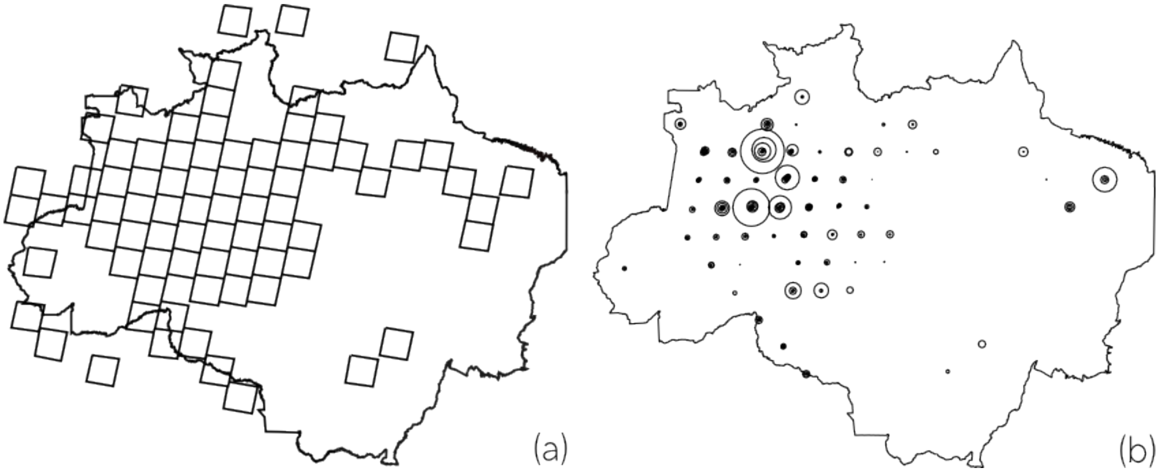

Supplementary Figure 5. *Landsat images and blow-down distribution.* (a) Spatial distribution of 72 Landsat scenes with the occurrence of blow-downs from the total 136 surveyed scenes of the Brazilian Amazon[26]. (b) The area of blow-down disturbance is proportional to the size of the circles. Landsat images with blow-downs outside of the Brazilian Amazon border were omitted from the spatial point analysis.

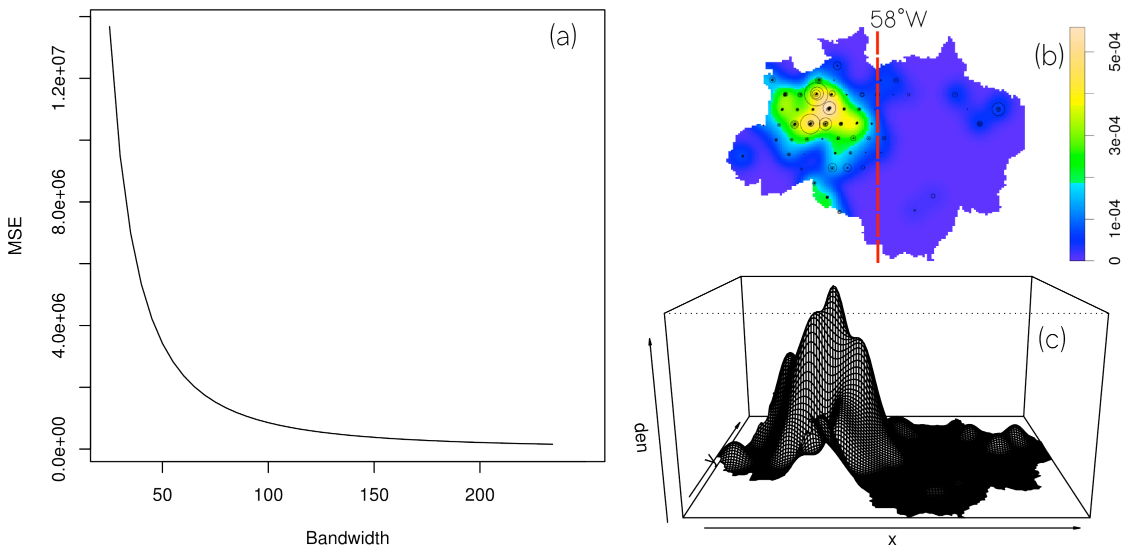

Supplementary Figure 6. *Kernel bandwidth distribution.* (a) Mean square error (MSE) of the Gaussian kernel smoothing algorithm[28,32] from the spatial distribution of 330 blow-downs data[26]. (b) The bandwidth with smaller MSE around 200 km is the less biased bandwidth for this spatial data. (c) East-West perspective graph of the intensity of blow-downs in the Amazon produced by a smoothing kernel interpolation.

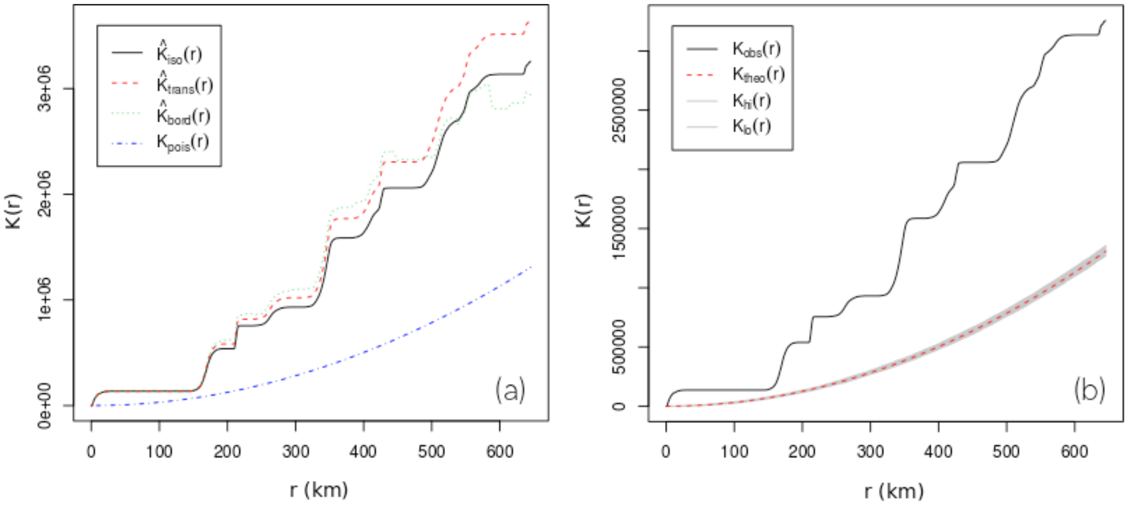

Supplementary Figure 7. *K-function distribution of the spatial patterns of blow-downs.* (a) *K*-function and simulated envelops of the spatial distribution of 330 blow-downs[26]. (b) Monte Carlo simulation (*T*=1000) of the *K*-function[28]. Color lines in *a* are the theoretical Poisson *K(pois)* of *K*-function in blue and the border-corrected estimate *K(bord)* in green, translational-corrected estimate *K(trans)* in red and the original Ripley isotropic correction *K(iso)* in black. Color lines in *b* are the original *K*-function[28] in black and red dash lines with upper and lower envelops in grey. The graph suggests that for all spatial simulations[32] the occurrences of blow-downs are clustered significantly.

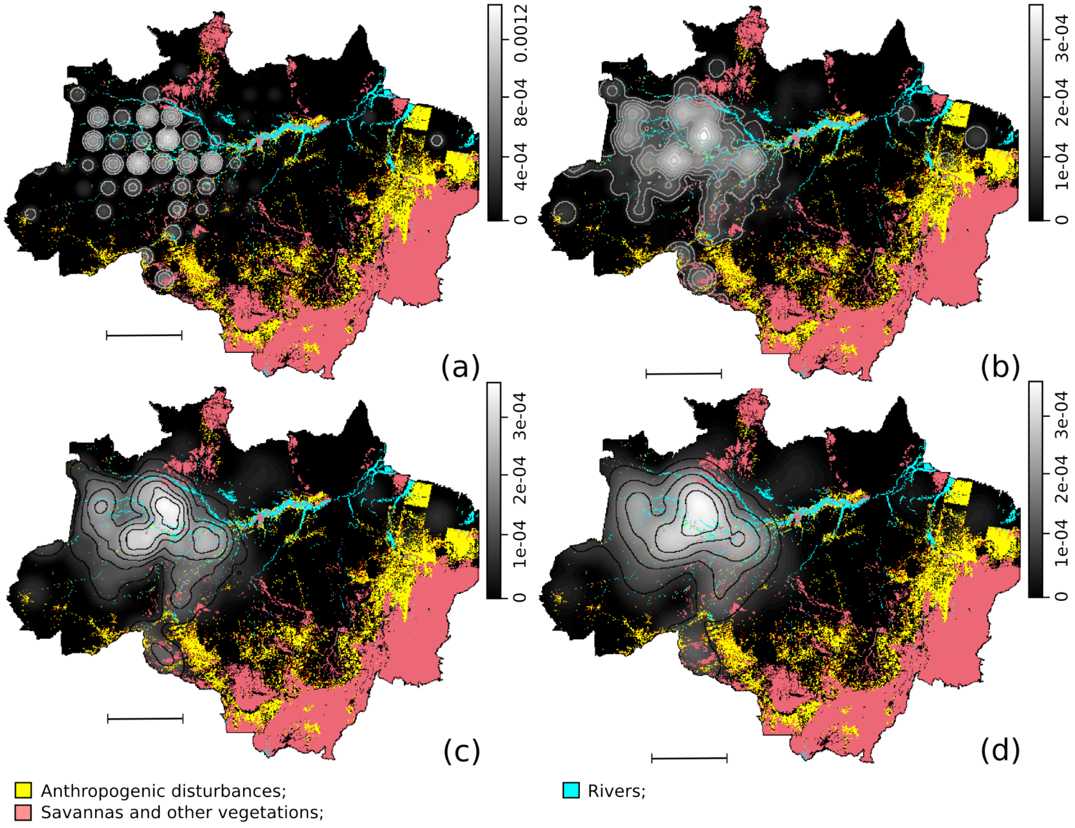

Supplementary Figure 8. *Clustering of large disturbances blow-downs in the Brazilian Amazon.* Blow-down clusters modeled with Kernel bandwidth of (a) 100, (b) 150, (c) 200 and (d) 250 km. Spatial patterns of blow-downs overlaid on a land-use and vegetation map produced by the Brazilian Space Agency INPE[18]. Color bar is the intensity of large disturbances in the Amazon (number of blow-downs per km^2). Legend of scale-bar for all maps of blow-down density is 500 km.

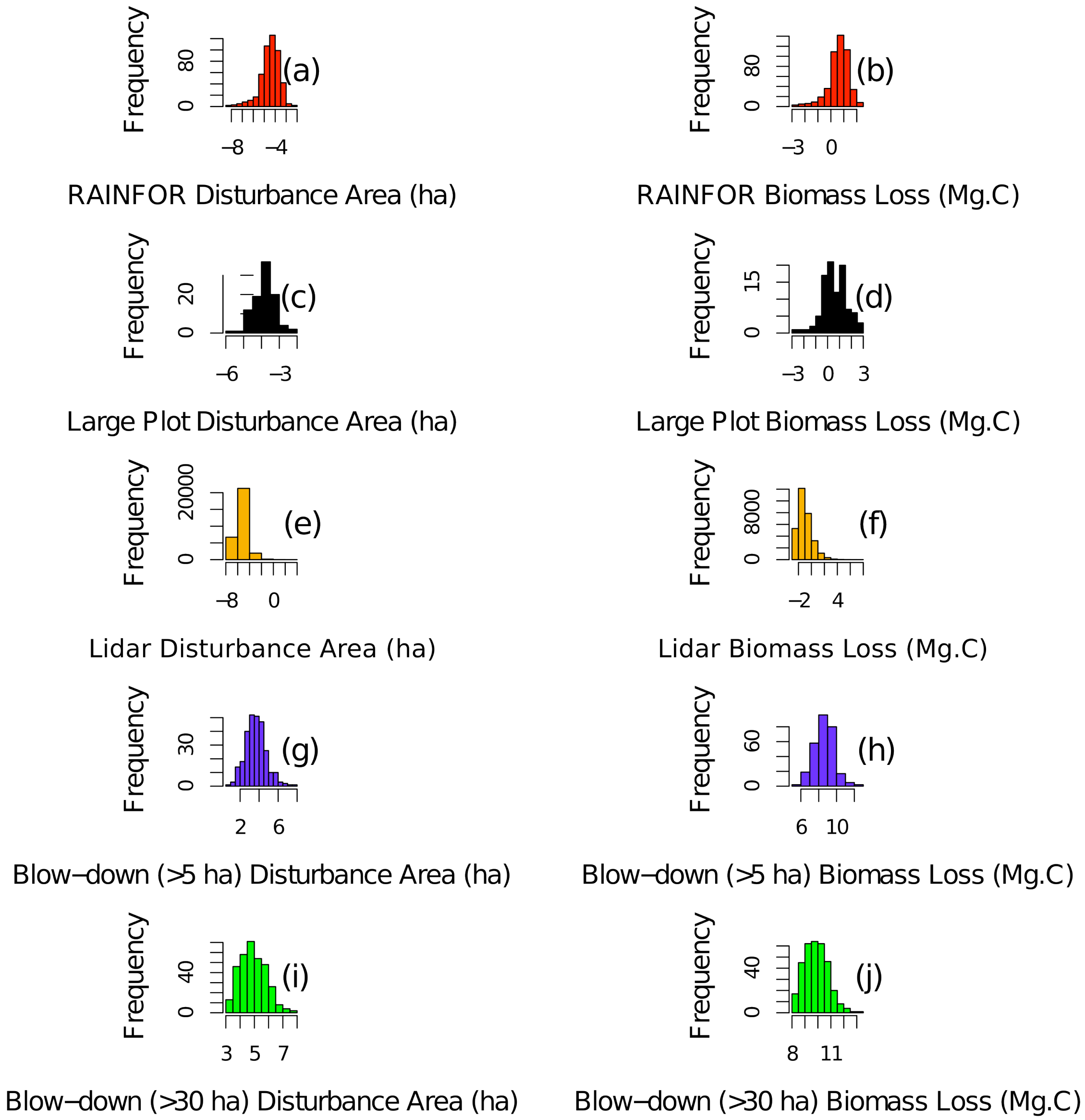

Supplementary Figure 9. *Frequency distributions (in log-scale) of area and biomass loss from five sources of natural disturbance data sets.* Small disturbances: (1) in red (a-b), forest plot inventories (n=484 censuses of 135 * 1ha plots[10]) distributed over the Amazon and (2) in black (c-d), 96 tree-fall gaps from two large forest inventory plots (total area 167 ha) in the Tapajós National Forest[23]. Intermediate disturbances: (3) in orange (e-f), small and intermediate disturbances from 48,374 ha of lidar[24] data in southern Peru. Large disturbances: (4) in blue (g-h), 279 blow-downs bigger than 5 ha from an East-West mosaic of 27 Landsat scenes of the Amazon[26]; and (5) in green (i-j), 330 blow-downs greater than 30 ha from 136 Landsat scenes in the Brazilian Amazon[25].

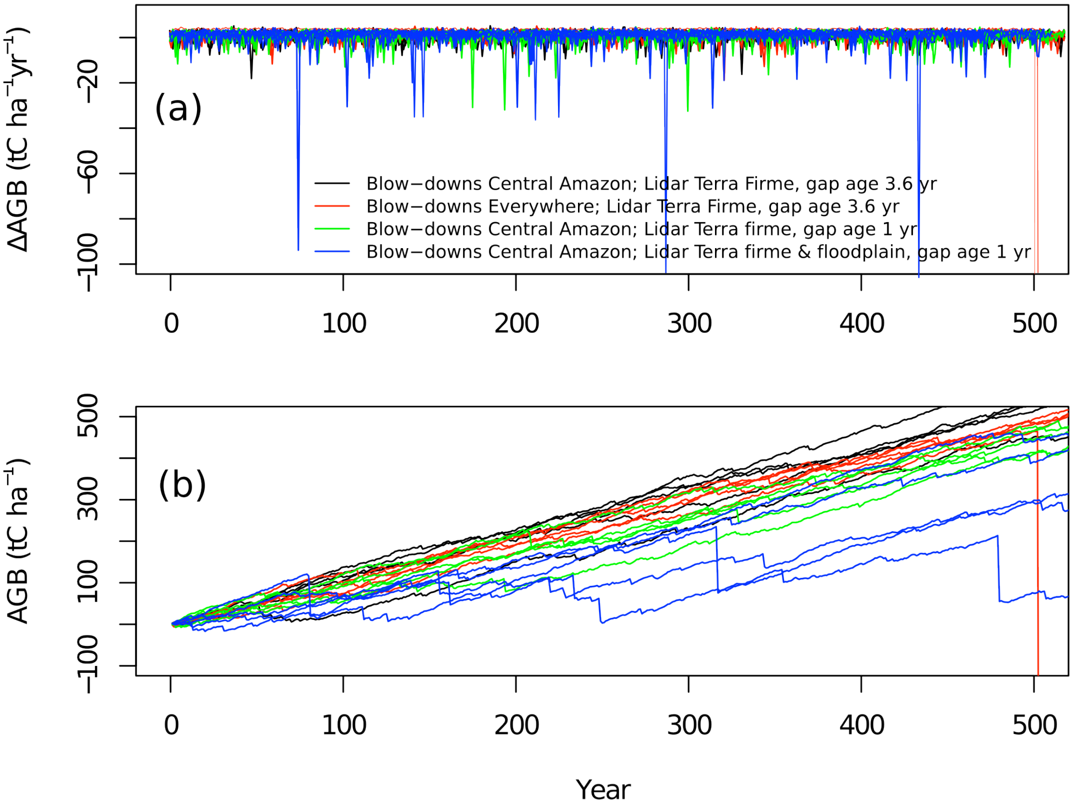

Supplementary Figure 10. *Simulations of the Amazon aboveground biomass change.* Simulations using the full frequency distribution of natural disturbance (small, intermediate and large-scale disturbances) assuming several scenarios of blow-downs occurrence and ages of tree-fall gaps from lidar data. Prediction examples of (a) the mean mass balance ΔAGB for annual time-steps and (b) mass balance trajectory of $AGB(year = N) = \sum_{i=1}^{N} \Delta AGB(i)$ for a few members of the sample are presented.

Supplementary Tables

Supplementary Table 1. *Statistical summary of all data sets used to estimate the full frequency spectrum of disturbance over the Amazon.* 484 censuses of 135 ~1 ha plots distributed over the Amazon[9,10,21,22], 48,374 ha of tropical forest sampled in Southern of Peru by airborne lidar[24], 96 tree-fall gaps in a167 ha plot in East central Amazon[23], 279 blow-downs ≥ 5 ha detected in 27 Landsat scenes[25] and 330 large disturbances ≥ 30 ha inspected in 137 Landsat images[26]. Minus sign denotes biomass losses.

Statistic summary	RAINFOR (484 censuses)	167 ha plot	Airborne lidar	Blow-downs in 27 images	Blow-downs in 136 images
Disturbances Class Size	Small	Small	Intermediate	Large	Large
Raw data for modeling	484	96	30,130	279	330
**Min. disturbance area (ha)**	**0.0003**	**0.003**	**0.002**	**5**	**30**
**Max disturbance area (ha)**	**0.09**	**0.13**	**9.48**	**2,223**	**2,651**
Mean disturbance area (ha)	0.016	0.026	0.009	79	213
Median disturbance area (ha)	0.013	0.022	0.003	37	123
SD of disturbance area (ha)	0.012	0.018	0.079	179	279
Sum of disturbance area (ha)	7.53	2.51	294.50	21,931	70,421
**Min. biomass loss (Mg C)**	**-0.055**	**-0.061**	**-0.1**	**-324.9**	**-3,068**
**Max biomass loss (Mg C)**	**-11.69**	**-19.90**	**-1,162**	**-389,131**	**-463,876**
Mean biomass loss (Mg C)	-2.32	-3.05	-1.04	-12,091	-30,198
Median biomass loss (Mg C)	-1.99	-1.63	-0.29	-5,239	-17,672
SD of biomass loss (Mg C)	-1.61	-3.61	-9.75	-31,347	-42,893
Sum of biomass loss (Mg C)	-1,126	-293.67	-31,474	-3,373,601	-9,965,230

Supplementary Table 2. Summary of Amazon forest simulator results and statistical significance of simulated mean aboveground biomass gains for a range of extreme scenarios. We analyze three cases of large-disturbance blow-downs[25,26], (the large-end tail of the disturbance frequency distribution): observed (*i*) no large disturbance events , (*ii*) only in central Amazon (~20% of the Amazon region), (*iii*) everywhere in the Amazon with the same frequency of events as in the Central Amazon (i.e. 5 times more frequent than detected). For intermediate-range disturbances occurring across the entire Amazon region distributed according to lidar surveys[24] (plots 1,4,5 and 12) of depositional-floodplain (DFP) forests (15,178 ha), and assuming an extreme case of a mean gap age of only 1 year. We also assumed an mean mass gain[8,10,11] of 2.75 Mg C ha^{-1} yr^{-1}. The simulator of forest mortality is based on the frequency distribution of disturbance area. To convert area losses to biomass losses we assumed a forest mass density of 170 Mg C ha^{-1} for all simulations, nearly 50% greater than the actual biomass density in the lidar landscape used to estimated intermediate disturbance dynamics[8,11]. Assessment of each scenario is based on a set of 10^9 annual equivalent samples. Significance is assessed with a *t*-test considering $t_{sim} = (dM/dt)/(\sigma/\mathrm{sqrt}(N))$ where *dM/dt* is ensemble mean mass gain, σ the standard deviation of the mass gain distribution and *N* the number of observations. For *N* we use either conservatively *N =135* the total number of observational plots or *N =1545*, the total number of plot census years reflecting the stochastic nature of disturbance and therefore the near independence of plot results from year-to-year. Gain results are statistically significant at the 95% level if $t_{sim} \geq t_{\{0.975,N=135\}} \approx t_{\{0.975,N=1545\}}=1.96$ and at the 99% level if $t_{sim} \geq t_{\{0.995,N=135\}} \approx t_{\{0.995,N=1545\}} = 2.58$.

Assumed annual mean mass gain[8,10,11]: 2.75 Mg C ha^{-1} yr^{-1} and intermediate-scale disturbances[13,20] modeled with:

Intermediate-Scale Disturbances	Large-Scale Blow-downs[25,26]		
Lidar data[24] from terra firme and floodplains (gaps age[30] ~ 1 yr old)	None	Central Amazon	All Amazon Region
dM/dt*	0.66	0.66	0.65
σ*	9.76	10.89	14.68
t_{obs}(N=135)	0.79	0.70	0.51
t_{obs}(N=1545)	2.65	2.38	1.74

Supplementary Methods

Data integration We quantified the frequency distribution of small[17,33–35] and large disturbances[25–27,36] from several sources of data. Our data ranges from permanent tree plots[9,10,21–23] to satellite[25,26] or airborne lidar[24] data (Supplementary Table 1). We quantify not only data of disturbance area, but also the aboveground biomass loss in Mg C associated with these events.

A flowchart summarizes all processing steps used to harmonize the data of natural disturbances over the entire Amazon region (Supplementary Figure 1). Five data sources were used to estimate disturbances: at small-scale (1) data set from two large plots[23] (167 ha) in Tapajós region, and (2) 484 repeat censuses of the tropical forest network[10,21,22,37]; at intermediate: (3) lidar from Southern Peruvian Amazon[24] (48,374 ha); and at large-scale: (4) blow-downs > 30 ha (n=330) covering the entire Brazilian Amazon, and (5) fine resolution blow-downs > 5 ha (n=279) covering a East-West Amazon forest region transect. Because each data source was collected and produced in different ways, we applied several intermediate steps to estimate and normalize the data. Our final goal was to use the probability distribution of area and biomass loss of natural disturbances to understand the trajectory of the Amazon forest carbon balance.

RAINFOR plots We used the extensive historical data set of the RAINFOR plots[4,8–10,21,22,37,38] based on net changes in biomass (Mg C ha^{-1} yr^{-1}) which include two aboveground biomass flux terms[39,40]: biomass gain (from tree growth and recruitment) and biomass loss (from tree mortality). The biomass loss from these plots was assessed to provide information of tree mortality across the Amazon region. Those plots are typically 1 ha in size and measurement details have been described elsewhere[8,10,21,22,38]. The available, published RAINFOR data (135 plots[10]) cover a total area of 226.2 ha with a mean total monitoring period of 11.3 years. Aboveground biomass and biomass dynamics were estimated from tree diameter and wood density (based on species identity) using a

published allometric equation[41]. Mortality rates have been corrected for census-interval effects[42]. The RAINFOR dataset used for this analysis has few if any plots within the large blow-down zone delineated in this paper, so presumably they cannot be recovering from large blow-downs.

Translating biomass loss measured in RAINFOR plots to disturbance area The RAINFOR network does not record disturbance area - but biomass losses due to mortality events – thus here we estimated the area of those disturbances associated with the biomass loss as gap area of a given plot = {mean biomass loss (ha^{-1})} ÷ {mean total stock of biomass (ha^{-1})}. This approach assumes that all biomass disturbances are linearly correlated with area of the disturbances which is a rough approximation[14]. Moreover, ground data of tree-fall gap disturbance areas and biomass losses from two large plots in Tapajós National Forest (54 and 114 ha, n=96 gaps) suggests that this relation is not fully linear (Supplementary Figure 3).

Large forest inventory plots data RAINFOR data[21] do not account for biomass losses (disturbances) that do not result in complete tree death (e.g. coarse woody debris (CWD) produced by partial crown-falls). To evaluate carbon losses including both complete and partial mortality, we installed and surveyed two large forest inventory plots[23] of 114 and 53 ha, in unmanaged forest area in the eastern central Amazon, Tapajós National Forest (TNF) (Fig. 1 and Supplementary Figure 3). The first plot was installed in 2008 and the second in 2009. The methodology to assess the biomass losses (CWD) inside of the gaps areas has been described elsewhere[23], with the main steps listed here:

1) We mapped all gaps in both large plots using the Runkle gap definition[43];

2) We defined the modes of gap-formation[17,33–35,43] based on the type of disturbance (partial or complete crown-fall, snapped bole-fall, and uprooted tree-fall);

3) We classified all gaps within two age classes (< 1 and ≥ 1 year old);

4) We measured the volume of all CWD for each gap identified in the field;

5) We used an allometric equation[44] to estimate woody biomass losses by fresh tree–falls and snapped bole falls while for gaps with partial crown-fall we recorded the diameters of all wood pieces greater than 10 cm and length of the woody material;

6) We classified the decomposition status[45–47] of all CWD into five decay classes - from freshest (class 1) to most rotten (class 5) material;

7) We used an average of CWD density measured for each decay class specifically developed for this site[46–48];

8) We calculated the sectional volume of each segment of CWD; and

9) We estimated the mass of CWD from the product of the volume of material and the respective density for the material class[46–48].

Biomass losses measured at the large forest inventory plots In the two large plots[23] (167 ha total area) we found 96 gaps. CWD amounts depended on the type of gap formation, crown-falls contained 0.11 Mg C ha^{-1} of CWD, snapped tree-falls 0.65 Mg C ha^{-1} and uprooted tree-falls 0.70 Mg C ha^{-1}. The flux of CWD caused by the gaps was 0.76 Mg C ha^{-1} $year^{-1}$. The average mortality of trees (DBH $\geq$ 10 cm) per gap was 6.5, resulting in a total of 596 dead individual trees (3.57 trees ha^{-1}; > 10 cm DBH) for the total surveyed area of 167 ha. From the total dead trees contained in the gaps of all ages, we estimated a mean annual tree mortality of 2.38 trees ha^{-1} $year^{-1}$.

Airborne lidar data To estimate the distribution of intermediate scale sized disturbances[13,20] (between 0.01 and 5 ha of opened area) we used a large collection of airborne lidar[24] data. Lidar (Light detection and ranging) is a remote sensing technology that measures distances by illuminating a target with a laser and analyzing the reflected light[49]. Recently, airborne lidar has been used to distinguish canopy gaps at large spatial scales[24,50,51], providing a unique opportunity to understand the frequency distribution of natural disturbances or tree-fall gaps.

We used lidar data collected by the Carnegie Airborne Observatory (CAO) Alpha System[52] (July 2009) in the Southern Peruvian Amazon[24]. The study was undertaken in the Madre de Dios watershed, in a region of well-known geologic and topographic variation in lowland forest close to the base of the Andes in Peru[24]. Briefly, the flights were conducted at 2000 m aboveground level at a speed of <95 knots. The lidar was operated with a 38-degree field of view and 50 kHz pulse repetition frequency, resulting in 1.1 m laser spot spacing[24]. We processed 4 blocks (Fig. 1g) covering a total of 48,374 ha. To compare gap-size frequency distributions among forests in the lowland Peruvian Amazon, lidar data were classified in each block by geologic composition and an empirical lidar digital terrain model of ~15 m height[24], resulting in two major types of forest areas[24]: "depositional-floodplain" (DFP) in 15,178 ha and erosional "terra firme" (ETF) in 33,196 ha (following the abbreviations in Asner et al[24]). Terra firme forests dominate Amazonia (RAINFOR[8–11,21]), we used the DFP data only for a sensitivity analysis of our forest simulator results to different forms of the Amazon disturbance frequency distribution.

To quantify all types of disturbances at landscape scale with lidar (i.e. from small 0.01 ha to intermediate scales 5 ha), the original lidar data points were processed[24] to generate raster images (pixel resolution = 1 m) of the digital canopy surface model (DSM) and digital terrain model (DTM). The DSM was based on interpolations of all first return points of the cloud data, where elevation is relative to a reference ellipsoid. The DTM was based on a 30 m x 30 m filter passed over each flight block and the lowest elevation estimate in each kernel was assumed to be the ground. Canopy heights (DCM) were estimated as the difference between the canopy surface model and the digital terrain model, i.e. as DCM=DSM-DTM[24] (Supplementary Figure 2).

Because lidar data analyses permit detection of all gaps extending from the top of the canopy to different heights aboveground[24,50,51] (i.e. 1-2 m tree height), we defined gaps in our lidar data using the ecological definition of Brokaw[33]: gaps in lidar digital canopy model are openings in the forest canopy extending down to an average height 2 m

aboveground (Supplementary Figure 2c,d). The minimum gap size considered was 20 m^2 (Supplementary Figure 2e).

Biomass loss associated with intermediate-scale disturbances To estimate biomass loss due to intermediate-size disturbance detected by lidar data (4 transects with a total of 48,343 ha, n=30,130 gaps) we used an allometric equation of biomass loss (Mg C) based on gap size of disturbances (ha) collected on the ground in two large forest inventories[23] (Supplemental Fig. S3). We used a minimum gap size area threshold of 20 m^2 of disturbance area to estimate CWD or biomass loss inside of tree-fall gaps areas detected by lidar. There are two reasons for using this approach: First, based on our previous analysis, measurable carbon loss was associated with a minimum gap area of ~20 m^2 or bigger (see Espírito-Santo et al., 2013[23]); and second whereas very small gaps (i.e. ~1 m^2) - where most of the sunflecks[31] occur - are probably more related with tree crown spacing[31] than with biomass carbon dynamics.

We estimated the necromass of small-intermediate disturbance areas detected by lidar[24] using a linear regression model of aboveground biomass loss (Mg C) as a function of gap-size area (ha) of central Amazon[23] (167 ha plot, n=96) (Supplemental Fig. S3). The resulting equation to estimate necromass from tree-fall gaps[23] has not been validated outside of our original study site and will slightly overestimate carbon loss in Peru where wood density averages 20% lower than in the Central Amazon[14].

Finally, the lidar datasets available currently are not repeat surveys and therefore only permit a snapshot of forest structure. To use these data to inform forest biomass dynamics evidently requires making a number of important assumptions about how these maps of gaps translate into forest disturbance rates. To ensure that our test of the hypothesis that the plot network effectively measures biomass change is conservative, our assumptions deliberately err on the generous side to the magnitude and frequency of intermediate area disturbance. Our assumptions will tend to overestimate the rate of formation of

intermediate-sized gaps, and therefore should overestimate their contribution to Amazon biomass dynamics. Notably, we assume

1) That the region surveyed is representative of Amazonia. In fact we know from our ground work that forests in western Amazonia have much faster biomass turnover and a greater proportion of tree death caused by exogenous disturbance than elsewhere (e.g., Phillips et al. 2004, Galbraith et al. 2013)[53,54].

2) That gap recovery rates are fast, with 50% closure within 3.6 years. This estimate is based on a transition matrix from Hubbell and Foster (1986)[30], indicating that at Barro Colorado Island, Panama, the 1-year transition probability for 5*5m gaps to non-gaps was 0.177. Alternatively, a study from French Guiana suggests a half-life of between 5 and 6 years (Fig 7 in Van de Meer and Bongers, 1996[55]), and with all gaps closing after about 15 years.

3) That gap recovery rates are independent of size within the 'intermediate' part of the spectrum. In practice, bigger gaps will take longer than small gaps to close so our approach is likely to overestimate the frequency of larger gap formation.

4) Our estimated gap formation rates are translated into biomass dynamics estimates assuming an AGB value of 170 Mg C ha-1. In fact, in 16 * one-hectare plots in the same region where the lidar data were taken, mean AGB is 119 Mg C ha-1. This assumption alone therefore results in overestimating the impact of intermediate biomass disturbances in south-western Amazonia by more than 40%.

Amazon forest area To scale up our results of natural forest disturbances from forest inventory plots[10,21,23], lidar[24] and satellite images[25,26], to the entire forest area of the Amazon, we used a land cover map with 250 m spatial resolution for all countries that are part of the Amazon tropical forest biome[18] (Supplemental Fig. S4). For the Brazilian Amazon region (approximately 60% of the entire Amazon) we used the land use map from the annual deforestation monitoring project (PRODES) of the National Institute for Space Research (INPE)[18] to separate old-growth forest from non-forest areas or recently deforested areas. PRODES has monitored tropical deforestation in Brazil over the last 30 years using historical Landsat images[56] using visual interpretation and digital image

processing[57]. To expand the land use map to South America (Pan-Amazon Project, unpublished data[18]), multi-temporal MODIS images of 250 m resolution were processed by the INPE Pan Amazon project[18] for the others regions and integrated to the PRODES database[56]. The land use map (Supplementary Figure 4) has the following categories: undisturbed forest, deforestation (general category of bare soils, secondary forests and burned areas), and other types of vegetation (savannas and grasses). According to this map the total area of undisturbed forest in northern South America is 6.8×10^6 km^2 covering the Amazon drainage region and the contiguous Andes and Guyana's regions[58] - the entire forested Brazilian Amazon is 3.5×10^6 km^2.

We used the entire Amazon region (6.8×10^6 km^2) to scale up all natural disturbances (Supplementary Table 1) recorded in our data. Considering that most blow-downs are concentrated in Central Amazon, we assumed that large disturbances cover 1/5 of the total area of our entire domain of Amazon forests (see also Tab. 1 for more details).

Basin-wide large disturbance data We developed a spatially explicit analysis of large disturbances (blow-downs) in the Brazilian Amazon tropical forest biome based on extensive samples of Landsat satellite images (30 m). We assessed the occurrence and spatial distribution of 330 events of large disturbances or blow-downs ($\geq$ 30 ha) during the period from 1986 to 1989 based on 137 Landsat images[28,32] (Supplementary Figure 5) using the original raw data from the first study that described the occurrence of blow-downs in the Amazon[26].

We also analyzed the occurrence and spatial distribution of 278 large forest disturbances ($\geq$ 5 ha) from 1999 to 2001 apparently caused by severe storms in a mostly unmanaged portion of the Brazilian Amazon using 27 Landsat images and digital image processing[25].

Spatial distribution of large disturbances Previous analyses of large disturbances showed that blow-downs are extremely rare in Eastern Amazonia[25,26]. To account for clustering of large disturbances in the Amazon we reanalyzed the original data of large natural disturbances from Brazil[26] using a spatial point analysis (SPA)[25]. A SPA consists

of a set of points (s_1, s_2, …, s_N) in a defined study region (R) divided into sub-regions ($A \subseteq R$). $Y(A)$ is the number of events in sub-region A. In a spatial context, the number of points can be estimated by use of their expected value $E(Y(A))$, and covariance COV ($Y(A_i)$, $Y(A_j)$), given that Y is the event number in areas A_i and A_j. The intensity of an event $\lambda(s)$ is the frequency per area of points of a specific location s, where ds is the area of this region, i.e. $\lambda(s) = \lim_{ds \to 0} \left\{ \frac{E(Y(ds))}{ds} \right\}$. Because SPA only requires the spatial location of each event, we used the center of each classified blow-down in the Landsat images. We used a Gaussian algorithm (kernel smoothing) with bandwidths between 100 and 250 km to calculate the smooth intensity field from our data. The minimum mean square error (MSE) of the Gaussian kernel smoothing algorithm[28,32] revealed that the bandwidths ~ 200 km (Supplementary Figure 6) is the most indicated to estimate the intensity of blow-downs in the Amazon. The probability density function k of Ripley[28] also suggests that large-scale disturbance blow-downs in the Amazon are strongly clustered[25] for the tested bandwidths (Supplementary Figure 7).

To determine the spatial distribution of blow-down over the entire region of Brazilian Amazon excluding the regions of intense land-use activities[1,59] (i.e. deforestation and fire) and other types of vegetation (i.e. savannas and sand forests) we used a land-use map (Pan-Amazon Project, unpublished data[18]) as described before. We excluded most of the anthropogenic disturbances caused by fires, but probably we did not remove some areas of undisturbed forests affected by the natural dynamics of fires (i.e. transitional regions of forest and savannas). Natural fires are expected to play a role in tree mortality, so future efforts should attempt to understand the scale and impact of natural fires on tree mortality in the Amazon[14].

The overlay of our most recent spatial grid of blow-downs (data from Nelson et al. 1994[26]) modeled with different kernel bandwidth[28] (100, 150, 200 and 250 km) from our SPA model confirmed that most large disturbance blow-downs in the Amazon are far from the deforestation arc. Spatial patterns of clustering of blow-downs are influenced by

the choice of kernel bandwidth sizes (Supplementary Figure 8). However, the bandwidth with smaller MSE[28] (200 km, Supplementary Figure 6) seems to be the most appropriate to resent the spatial pattern of blow-downs in the Brazilian Amazon. Yet, independent of the bandwidth choice, the analysis shows the same main spatial patterns of blow-downs. The density of large-scale blow-downs in the Amazon increases from East to West and South to North with the epicenter blow-downs around of Purus River region[25,26].

Biomass loss of large-scale disturbances For all events of large-scale blow-downs[25,26] (n=609, sum of blow-down records of Nelson et. al, 1994[26] and Espírito-Santo et al., 2010[25]), we estimated the biomass loss as the product of disturbance area and its respective mean aboveground biomass extracted from the regional map of biomass stock of the Amazon[5] region with 1 km^2 spatial resolution (Fig. 1). We assume 100% mortality in areas of blow-downs[14,25,26,36,60]. We anticipate that this mortality rate overestimates carbon loss[12,14,20], and so provides an upper bound estimate of the significance of large natural disturbances[12,13] to old-growth forest carbon accumulation rates. Although not perfect, we provide the closest estimation of biomass loss by blow-downs based on class size of large-scale disturbances and the spatial gradient of biomass distribution in the Amazon[5].

Disturbance area and biomass loss From tree-fall gaps to landscape blow-downs we provide the statistics of natural disturbances data for the various data sets in terms of area and biomass loss (Supplementary Figure 9). Because several data have the frequency distribution concentrated over small range of the data (skewed frequency distribution), we also provide the histograms of disturbances in a log transformation for a better visualization. In general, the frequency distributions of the different types of disturbances do not overlap completely (Supplementary Figure 9) and our data set covers all scales of natural disturbances.

Assessing uncertainties of the natural disturbance Our general approach to quantify uncertainties is to use simulation scenarios that bracket the likely range of outcomes associated with various specific sources of uncertainty.

Uncertainties of our analysis are associated with combining datasets to obtain a region-wide disturbance size frequency distribution and simulation results based on such distributions. In order to address the problem of combining data sets, we note that the methods for detecting disturbances used in this study are suitable for different spatial scales (e.g. Landsat suitable to detect large blow-downs) and mostly do not overlap with respect to disturbance size range. If the datasets do not overlap we scaled them to the full region by multiplication with Amazon forested area-to-area probed before combining them (forests censuses, lidar data, Landsat imagery). In this case there is no need to take into account uncertainties for the combination (not for assessing uncertainties related to the simulations though – which we address as explained under the simulation Table 1). Where there is overlap in the size range covered by different datasets (relevant only to different plot data) obtained with different methods we combined the data by weighting inversely with area probed.

To address uncertainties related to our simulation we first briefly recapitulate our data sets and their spatial coverage. For smallest disturbances monitored by forest censuses (RAINFOR data[10]) spatial coverage is good with plots distributed well along the major axes of variation[21] (soil fertility, dry season length, El Nino influence) (Fig 1a), but missing the core region of large blow-downs. Largest disturbances are observed with Landsat imagery[25,26] which cover approximately 60 % of the Amazon forest region and the dataset includes 609 blow-downs (sum of blow-down records of Nelson et. al, 1994[26] and Espírito-Santo et al., 2010[25]). Spatial coverage is thus also representative for most of the Amazon region. In contrast, the lower end of the intermediate range is covered by data from a 114 and a 53 ha plot[23] in Tapajós National Forest and by lidar data[24] from southern Peru (Madre de Dios region). Thus the observations of the intermediate range are spatially biased (Fig. 1b,c).

Uncertainties to be addressed with a range of scenarios are thus due to:

1) *Spatial coverage*. As mentioned above, in contrast to small scale and largest scale disturbances lidar data[24] covering a substantial part of the intermediate range are only from one part of Western Amazonia. *We address this with a scenario whereby we assume the disturbance size distribution of the intermediate range to be the one obtained when combining the lidar data from terra firme and floodplains, a dramatic although unrealistic case;*

2) *Methodological issues*. For forest censuses these include uncertainties in allometries which although non-trivial are unlikely to have much impact here(see for example Feldpausch et al. 2012[61]). A concern with lidar data is the question how long a gap (or disturbance) is detectable by lidar. *We address this issue by running our simulator assuming either (a) a detectability time of 1 year or (b) a detectability time of 3.6 year respectively. The 3.6 years are chosen based on observation of gap closure in 50 ha plot of Barro Colorado Island from Hubbell and Foster 1986*[30]. Gap closure varies regionally, as data from French Guiana suggest half-lives of small forest gaps in excess of 5 years[55]. The 1-year detectability scenario is thus probably biologically unrealistic.

3) *Dependence of disturbance size frequency distribution on our given data sample*. We have calculated the uncertainties associated with calculating histograms formally and uncertainties are mostly not large with exception of the largest scales; *we analyze the effect of this source of uncertainty with the following scenarios: (a) assumption of occurrence of largest scale disturbances throughout the region (i.e. not just in the Central Amazon), (b) the standard – in our view most likely case - and (c) omission of largest blow-downs altogether across the entire region.* In light of extensive available data from two studies over two separate time periods using different analysis methods[25,26], we assert that both the full region disturbance and no disturbance scenarios are improbable, although in fact RAINFOR plots are not in the remote higher frequency blow-down zone.

4) *Dependence on observed growth statistics based on RAINFOR forest censuses*. We address this by centering growth (*G*) around the Amazon region mean of 2.50 Mg C ha^{-1} yr^{-1} and alternatively the Western Amazon region mean of 2.75 Mg C ha^{-1} yr^{-1} (see Gloor et al.[10]).

5) *Central Amazonia (where largest blow-downs are concentrated) versus rest of the Amazon region.* To address this issue we use the same scenarios as described under (3).

The results of the various simulation scenarios are summarized in Table 1 (see main manuscript for more details) and Table S2 (an extreme scenario that assumes the largest blow-downs occurring not only in Central Amazonia but throughout the Amazon regions and intermediate disturbances occurring at a rate that greatly over-represents the importance of floodplain forests). Sample trajectories for a range of scenarios are shown in Supplementary Figure 10.

Supplementary References

31. Chazdon, R. L. & Pearcy, R. W. The Importance of sunflecks for forest understory plants - photosynthetic machinery appears adapted to brief, unpredictable periods of radiation. *Bioscience* **41,** 760–766 (1991).

32. Baddeley, A. & Turner, R. Spatstat: an r package for analyzing spatial point patterns. *J. Stat. Softw.* **6,** 1–42 (2005).

33. Brokaw, N. V. L. The definition of treefall gap and its effect on measures of forest dynamics. *Biotropica* **14,** 158–160 (1982).

34. Fraver, S., Brokaw, N. V. L. & Smith, A. P. Delimiting the gap phase in the growth cycle of a Panamanian forest. *J. Trop. Ecol.* **14,** 673–681 (1998).

35. Van der Meer, P. J., Bongers, F., Chatrou, L. & Riera, B. Defining canopy gaps in a tropical rain-forest - effects on gap size and turnover time. *Acta Oecologica - Int. J. Ecol.* **15,** 701–714 (1994).

36. Chambers, J. Q. *et al.* Hyperspectral remote detection of niche partitioning among canopy trees driven by blowdown gap disturbances in the Central Amazon. *Oecologia* **160,** 107–117 (2009).

37. Phillips, O. L., Lewis, S. L., Baker, T. R., Chao, K. J. & Higuchi, N. The changing Amazon forest. *Philos. Trans. R. Soc. B-biological Sci.* **363,** 1819–1827 (2008).

38. Baker, T. R. *et al.* Variation in wood density determines spatial patterns in Amazonian forest biomass. *Glob. Chang. Biol.* **10,** 545–562 (2004).

39. Chave, J. *et al.* Assessing evidence for a pervasive alteration in tropical tree communities. *PLoS Biol.* **6,** e45 (2008).

40. Pyle, E. H. *et al.* Dynamics of carbon, biomass, and structure in two Amazonian forests. *J. Geophys. Res.* **113,** G00B08 (2008).

41. Chambers, J. Q., Santos, J., Ribeiro, R. J. & Higuchi, N. Tree damage, allometric relationships, and above-ground net primary production in central Amazon forest. *For. Ecol. Manage.* **152,** 73–84 (2001).

42. Malhi, Y. *et al.* The above-ground coarse wood productivity of 104 Neotropical forest plots. *Glob. Chang. Biol.* **10,** 563–591 (2004).

43. Runkle, J. R. Gap regeneration in some old-growth forests of the eastern United States. *Ecology* **62,** 1041–1051 (1981).

44. Brown, S. *Estimating biomass and biomass change of tropical forests: a primer*. (Food and Agriculture Organization of the United Nations (FAO), Rome, Italy, 1997).

45. Harmon, M. E., Whigham, D. F., Sexton, J. & Olmsted, I. Decomposition and mass of woody detritus in the dry tropical forests of the northeastern Yucatan Peninsula, Mexico. *Biotropica* **27,** 305–316 (1995).

46. Keller, M., Palace, M., Asner, G., Pereira, R. & Silva, J. N. Coarse woody debris in undisturbed and logged forests in the eastern brazilian Amazon. *Glob. Chang. Biol.* **10,** p784–795 (2004).

47. Palace, M., Keller, M., Asner, G. P., Silva, J. N. M. & Passos, C. Necromass in undisturbed and logged forests in the brazilian Amazon. *For. Ecol. Manag.* **238,** 309–318 (2007).

48. Palace, M., Keller, M. & Silva, H. Necromass production: studies in undisturbed and logged Amazon forests. *Ecol. Appl.* **18,** 873–884 (2008).

49. Means, J. E. *et al.* Use of large-footprint scanning airborne lidar to estimate forest stand characteristics in the western cascades of Oregon. *Remote Sens. Environ.* **67,** 298–308 (1999).

50. Kellner, J. R. & Asner, G. P. Convergent structural responses of tropical forests to diverse disturbance regimes. *Ecol. Lett.* **12,** 887–897 (2009).

51. Boyd, D. S., Hill, R. A., Hopkinson, C. & Baker, T. R. Landscape-scale forest disturbance regimes in southern Peruvian Amazonia. *Ecol. Appl.* **27,** 1588–1602 (2013).

52. Asner, G. P. *et al.* Carnegie Airborne observatory: in-flight fusion of hyperspectral imaging and waveform light detection and ranging (wLiDAR) for three-dimensional studies of ecosystems. *J. Appl. Remote Sens.* **1,** 1–21 (2007).

53. Phillips, O. L. *et al.* Pattern and process in Amazon tree turnover, 1976–2001. *Philos. Trans. R. Soc. Biol. Sci.* **359 ,** 477–491 (2004).

54. Galbraith, D. *et al.* Residence times of woody biomass in tropical forests. *Plant Ecol. & Divers.* **6,** 139–157 (2013).

55. Van der Meer, P. J. & Bongers, F. Formation and closure of canopy gaps in the rain forest at Nouragues, French Guiana. *Vegetatio* **126,** 167–179 (1996).

56. Tardin, A. T. *et al.* *Levantamento de areas de desmatamento na Amazônia legal através de imagens do satélite Landsat*. **Inpe-1411,** 14 (Instituto Nacional de Pesquisas Espaciais, 1979).

57. Shimabukuro, Y. E. & Smith, J. A. The least-squares mixing models to generate fraction images derived from remote sensing multispectral data. *IEEE Trans. Geosci. Remote Sens.* **29,** 16–20 (1991).

58. Ter Steege, H. *et al.* Continental-scale patterns of canopy tree composition and function across Amazonia. *Nature* **443,** 444–447 (2006).

59. Houghton, R. A. Aboveground forest biomass and the global carbon balance. *Glob. Chang. Biol.* **11,** 945–958 (2005).

60. Negrón-Juárez, R. I. *et al.* Widespread Amazon forest tree mortality from a single cross-basin squall line event. *Geophys. Res. Lett.* **37,** L16701 (2010).

61. Feldpausch, T. R. *et al.* Tree height integrated into pan-tropical forest biomass estimates. *Biogeosciences* **9 ,** 3381–3403 (2012).
